# Endoscopic ultrasound-guided lauromacrogol embolization for suspected Zollinger–Ellison syndrome: a novel approach

**DOI:** 10.1055/a-2646-1779

**Published:** 2025-07-25

**Authors:** Fangfang Guo, Bo Li, Lingfang Shi, Hongtan Chen

**Affiliations:** 1 Department of Gastroenterology, The First Affiliated Hospital, Zhejiang University School of Medicine, Hangzhou, China; 2 223524 Department of Gastroenterology, Affiliated Hospital of Shaoxing University, Shaoxing, China

Zollinger–Ellison syndrome is a rare condition caused by gastrin-secreting neuroendocrine tumors, leading to severe gastric acid hypersecretion, peptic ulcers, and diarrhea 1 . Accurate diagnosis remains challenging, requiring characteristic clinical symptoms, hypergastrinemia, and tumor localization through advanced imaging techniques 2 . Though conventional treatments include high-dose proton pump inhibitors, somatostatin analogs, or surgery 3 , we present a groundbreaking case of suspected Zollinger–Ellison syndrome successfully managed with endoscopic ultrasound (EUS)-guided lauromacrogol embolization, offering a minimally invasive yet highly effective therapeutic alternative.

A 58-year-old woman presented with a 5-month history of refractory watery diarrhea (up to 20 episodes daily) and weight loss of 10 kg. Previous hospitalizations showed partial response to octreotide but no improvement with antispasmodics or probiotics. Laboratory investigations revealed markedly elevated fasting serum gastrin (913.0 pg/mL). Upper endoscopy demonstrated multiple atypical duodenal and jejunal ulcers ( Fig. 1 ), and ¹⁸F-octreotide positron emission tomography and computed tomography identified a 1.5-cm hypermetabolic nodule (maximum standardized uptake value 77.7) in the gastric antrum, suggestive of a gastrinoma. Subsequent EUS evaluation confirmed a hypoechoic, hypervascular submucosal mass originating near the pyloric orifice ( Fig. 2 ). Although EUS-guided fine-needle aspiration yielded no malignant cells, the collective clinical, biochemical, and radiological findings strongly supported a diagnosis of Zollinger–Ellison syndrome.

**Fig. 1:**
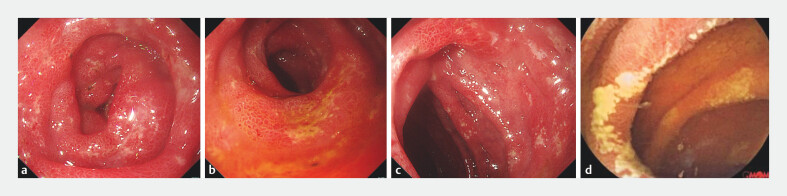
Gastroscopy and capsule endoscopy images of the patient. **a–c** Gastroscopy images showed multiple ulcer lesions in the duodenum. **d** Capsule endoscopy revealed atypical jejunal ulcers.

**Fig. 2:**
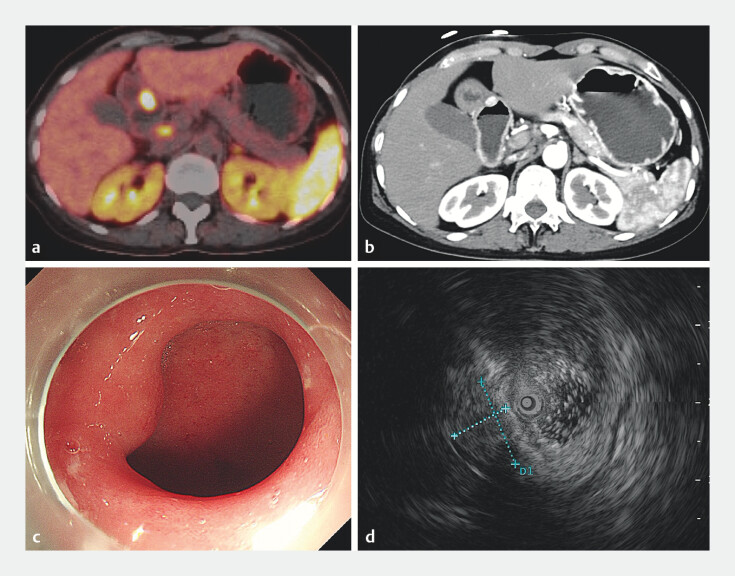
Imaging studies of the suspected tumor in the gastric antrum. **a, b** ¹⁸F-octreotide positron emission tomography and computed tomography (CT) ( **a** ) and contrast-enhanced CT ( **b** ) identified a 1.5-cm hypermetabolic nodule (maximum standardized uptake value 77.7) in the gastric antrum. **c** gastroscopy did not reveal any ulcer or submucosal tumor in the antrum. **d** Endoscopic ultrasound revealed a hypoechoic submucosal mass near the pyloric orifice.

Given the patient’s personal preference against invasive intervention, we performed EUS-guided embolization ( Video 1 ). A 22-gauge needle punctured the vessels feeding the lesion, followed by sequential injections of lauromacrogol. Post-procedural imaging confirmed complete occlusion ( Fig. 3 ). Remarkably, the patient’s symptoms resolved rapidly, and serum gastrin levels normalized upon discharge. At 4-month follow-up, she remained asymptomatic with no evidence of recurrence or procedure-related complications.

Endoscopic ultrasound-guided lauromacrogol embolization for suspected Zollinger–Ellison syndrome.Video 1

**Fig. 3:**
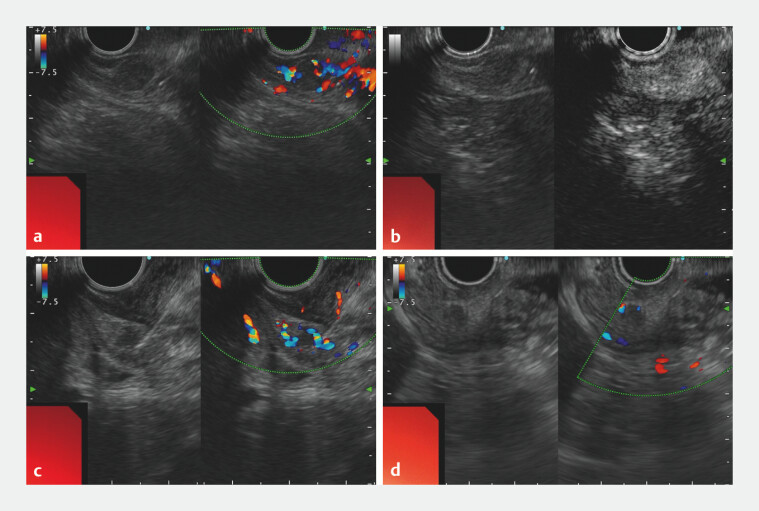
Endoscopic ultrasound (EUS)-guided lauromacrogol embolization for suspected gastrinomas in the gastric antrum. **a** EUS with color Doppler showed a hypoechoic, hypervascular submucosal mass originating near the pyloric orifice. **b** Contrast-enhanced harmonic EUS revealed intense arterial-phase enhancement. **c** A 22-gauge needle punctured the arteries feeding the lesion, followed by sequential injections of lauromacrogol. **d** EUS with color Doppler showed complete occlusion of target lesion.

EUS-guided lauromacrogol embolization represents an innovative and effective approach for the definitive ablation of suspected antral Zollinger–Ellison syndrome, offering a minimally invasive yet precise therapeutic option for small sporadic gastrinomas. Further prospective studies are needed to validate its long-term efficacy in managing functional neuroendocrine tumors.

Endoscopy_UCTN_Code_TTT_1AS_2AD

## Notes

Endoscopy E-Videos https://eref.thieme.de/e-videos
*E-Videos* is an open access online section of the journal *Endoscopy* , reporting on interesting cases and new techniques in gastroenterological endoscopy. All papers include a high-quality video and are published with a Creative Commons CC-BY license. Endoscopy E-Videos qualify for HINARI discounts and waivers and eligibility is automatically checked during the submission process. We grant 100% waivers to articles whose corresponding authors are based in Group A countries and 50% waivers to those who are based in Group B countries as classified by Research4Life (see: https://www.research4life.org/access/eligibility/ ). This section has its own submission website at https://mc.manuscriptcentral.com/e-videos .
